# Dyspepsia and gastric emptying in end-stage renal disease patients on hemodialysis

**DOI:** 10.1186/1471-2369-14-275

**Published:** 2013-12-14

**Authors:** Luiz Derwal Salles Junior, Paulo Roberto Santos, Armênio Aguiar dos Santos, Marcellus Henrique Loiola Ponte de Souza

**Affiliations:** 1 Sobral School of Medicine, Federal University of Ceará, Avenida Comandante Maurocélio Rocha Ponte, 100 - CEP 62.042-280, Sobral, CE, Brazil; 2 Department of Physiology and Pharmacology, Federal University of Ceará, Rua Coronel Nunes de Melo, 1127 – CEP 60.431-970, Fortaleza, CE, Brazil

**Keywords:** End-stage renal disease, Hemodialysis, Dyspepsia, Gastric motility, Delayed gastric emptying

## Abstract

**Background:**

Dyspepsia is common among end-stage renal disease (ESRD) patients and its association with delayed gastric emptying is not well established. We assessed the association of dyspepsia with gastric emptying time in ESRD patients undergoing hemodialysis (HD).

**Methods:**

Dyspepsia was assessed through the Porto Alegre Dyspeptic Symptoms Questionnaire (PADYQ). PADYQ’s scores ≥ 6 classified participants as dyspeptic. The octanoic acid breath test using ^13^carbon was employed to assess the gastric emptying time. Based on the test, time in minutes to metabolize the first half of the ^13^carbon in the test meal (t1/2) was calculated. Association of dyspepsia with gastric emptying time was tested by the correlation between PADYQ scores and t1/2, and also by comparing t1/2 between dyspeptics and non-dyspeptics.

**Results:**

There were 34 (68.0%) dyspeptic patients. Dyspepsia score was positively correlated with t1/2 (r = 0.366; p = 0.009). Dyspeptics had longer t1/2 compared to non-dyspeptics, respectively, 238.0 ± 92.9 versus 185.5 ± 45.5 minutes (p = 0.042).

**Conclusions:**

Delayed gastric emptying was associated with dyspepsia. Prokinetic medications could have a role in preventing or relieving dyspeptic symptoms among HD patients. Future research in larger samples is necessary to confirm this association.

## Background

The most common non-renal complaints in end-stage renal disease (ESRD) patients are gastrointestinal symptoms such as heartburn, constipation, diarrhea and dyspepsia [1]. Dyspepsia is highly prevalent and characterized by upper abdominal pain, nausea, vomiting, upper abdominal bloating, and early satiety [2]. The prevalence of dyspepsia among HD patients varies between 48% and 70% [3,4]. In the general population, dyspepsia has been shown to impair quality of life [5]. Nevertheless, dyspeptic symptoms are not widely investigated among patients treated by HD, as is usually the case in relation to cardiovascular disease, osteodystrophy and nutritional status.

Dyspepsia may have organic and functional causes. Like in the general population, functional dyspepsia is the most frequent among ESRD patients [6]. There are reports of the possible role of gastric emptying delay as a cause of dyspeptic symptoms in HD patients [3]. Gastroparesis is better recognized in diabetics. However, its presence is also high in all-cause ESRD, varying between 36% to 62% among patients undergoing chronic HD [3,7]. In these patients, delay in gastric emptying can be related to malnutrition and hormonal and electrolytic disturbances [8].

Dyspepsia can be easily evaluated through a questionnaire about characteristic symptoms, although gastric emptying time is not so easily assessed. The tools available to estimate gastric emptying time are: technetium-99 m scintigraphy (gold –pattern) [9]; time of appearance of acetaminophen in blood after its ingestion [10]; imaging studies using 3D ultrasonography and nuclear resonance [11,12]; the smart pill (which seems to be a practical and promising method) [13]; and octanoic acid breath test using ^13^carbon (with 89% sensitivity compared to gold-standard technetium-99 m scintigraphy) [14].

In our study, we looked for an association between dyspepsia and gastric emptying time in an experiment conducted with a sample of ESRD patients on maintenance HD.

## Methods

### Sample

Fifty ESRD patients were randomly selected from a total of 225 patients undergoing HD in a single dialysis center during October 2011, after excluding patients: (i) under 18 years old, (ii) with less than three months of maintenance HD, (iii) with a history of abdominal surgery, chronic obstructive lung disease or chronic hepatic disease, (iv) with gallstone by ultrasonography, and (v) with endoscopy showing gastric ulcer, inflammation or structural alterations. Written informed consent was obtained from all participants, and the study was approved by the ethics committee of Federal University of Ceará.

### Dyspepsia assessment

Dyspepsia was assessed using a validated Brazilian version of a standardized questionnaire named the Porto Alegre Dyspeptic Symptoms Questionnaire (PADYQ) [2]. PADYQ allows quantitative analysis of symptoms of dyspepsia not related to ulcer and in accordance with the Rome I Consensus. It contains 11 questions about the presence, frequency, duration and intensity of five symptoms: upper abdominal pain, nausea, vomiting, upper abdominal bloating, and early satiety. Subjects were asked about the occurrence of these symptoms in the previous 30 days. Maximum score of 44. A score ≥ 6 classified the subject as dyspeptic. The assessment was administered by a single interviewer who was not a member of the regular dialysis unit team.

### Gastric emptying time assessment

Patients were instructed to avoid smoking and eating food rich in C-4 plants, like corn (including baked goods made with cornmeal) and pineapples, in the week before the study. After a minimum of 10 hours of fasting and immediately before the intermediate weekly session of HD, all patients completed the octanoic acid breath test was performed [14]. They were instructed to eat a solid meal, consisting of a scrambled egg with the yolk labeled with 100 μg of ^13^carbon octanoic acid. After homogenizing the yolk, the egg white was added, beaten and baked. It was ingested with 60 g of white bread and 5 g of margarine during 1 to 5 min and followed immediately by 150 mL of water.

To collect the breath samples, the patients exhaled into closed aluminized plastic bags, before the test meal (baseline), and then at 15-minute intervals during 2 hours and then every 30 min for a further 2 hours. The patients were advised to remain seated and refrain from physical activity during the test [15].

The gastric emptying rate was defined by half-emptying time (t1/2). T1/2 is the time in minutes for the first half of the ^13^carbon dose in the test meal to be metabolized. The t1/2 was calculated as described by Ghoos et al. [14]. Accordingly, t1/2 of more than 200 minutes identified gastric emptying delay. Both the apparatus (IRIS II-^13^C-Breath Test System) and substrate (^13^C-octanoic acid) used here were supplied by Wagner Analysen Technik (Bremen, Germany).

### Patient data

The demographic data, length of time on dialysis, number of current medications, use of antacids and underlying etiology of ESRD were obtained from the renal unit medical records. The underlying renal disease was classified according to clinical criteria only. Body mass index was calculated as Kg/m^2^. All participants completed laboratory tests for serum creatinine, albumin, hemoglobin, calcium and phosphorus were performed. The dose of dialysis delivered was evaluated using a second-generation Kt/V equation by Daugirdas [16].

### Statistical analyses

Data are mean ± SD or percentage. Comparisons were performed by the Student-t and Mann-Whitney tests for continuous variables, respectively with or without normal distribution. Comparisons of frequencies were carried out by the Fisher test. The Pearson test, adjusted to traditional HD sample confounders (age, gender, diabetes, time on dialysis, hemoglobin, albumin and Kt/V), was used to test correlation between continuous variables. Statistical significance was considered to be a P value of < 0.05. All the statistical analyses were performed using the SPSS version 13.0 program package.

## Results

Our sample consisted of 36 (72%) men and 14 (28%) women, with mean age of 42.5 ± 16.6 years, undergoing maintenance HD for 32.4 ± 34.7 months. Primary renal diseases were glomerulonephritis in 17 (34%), hypertensive nephrosclerosis in 12 (24%), diabetes in 12 (24%), obstructive nephropathy in 6 (12%) and undetermined in 3 (6%). Patients had mean body mass index within normal range (22.4 ± 4.1).

The laboratory results were hemoglobin of 10.1 ± 2.0 g/dl, albumin of 4.1 ± 0.7 g/dl, calcium of 9.2 ± 1.0 mg/dl, phosphorus of 4.7 ± 1.4 mg/dl and Kt/V of 1.4 ± 0.2.

Thirty-four (68%) patients were dyspeptic (PADYQ score ≥ 6). Mean PADYQ scores were 2.5 ± 2.3 and 16.8 ± 6.4 among non-dyspeptics and dyspeptics, respectively. There were no significant differences in the demographic and laboratory variables between patients with and without dyspepsia (Table 1).

**Table 1:** Sample characteristics according to dyspepsia

**Variables**	**With dyspepsia**	**Without dyspepsia**	**P**
**Gender**			0.508
Male	23 (67.6)	13 (81.3)	
Female	11 (32.4)	3 (18.8)	
**Age**	39.6 ± 16.5	48.6 ± 15.5	0.075
**Etiology of ESRD**			
Glomerulonephritis	14 (41.2)	3 (18.8)	0.200
Hypertension	9 (26.5)	3 (18.8)	0.728
Diabetes	6 (17.6)	6 (37.5)	0.163
Obstructive uropathy	3 (18.8)	3 (18.8)	0.370
Undetermined	2 (5.9)	1 (6.3)	1.000
**Months on dialysis**	31.9 ± 30.7	33.5 ± 43.1	0.877
**Using antacids**	21 (61,7)	4 (25,0)	0.173
**Number of medications**	3.3 ± 0.9	3.6 ± 0.8	0.972
**Body mass index **(kg/m^2^)	22.3 ± 4,5	22.6 ± 3.3	0.897
**Creatinine** (mg/dl)	8.5 ± 2.9	8.1 ± 2.4	0.611
**Hemoglobin** (g/dl)	9.8 ± 2.1	10.9 ± 1.5	0.075
**Albumin** (g/dl)	4.1 ± 0.7	4.2 ± 0.8	0.571
**Calcium** (mg/dl)	9.1 ± 1.0	9.5 ± 1.0	0.155
**Phosphorus** (mg/dl)	4.9 ± 1.5	4.2 ± 1.1	0.115
**Kt/V**	1.4 ± 0.2	1.4 ± 0.2	0.965

Data are means ± SD, or percentages (in parentheses).

ESRD, End-stage renal disease.

The breath test was well tolerated by all participants and did not cause any adverse reactions.

**Figure 1:**
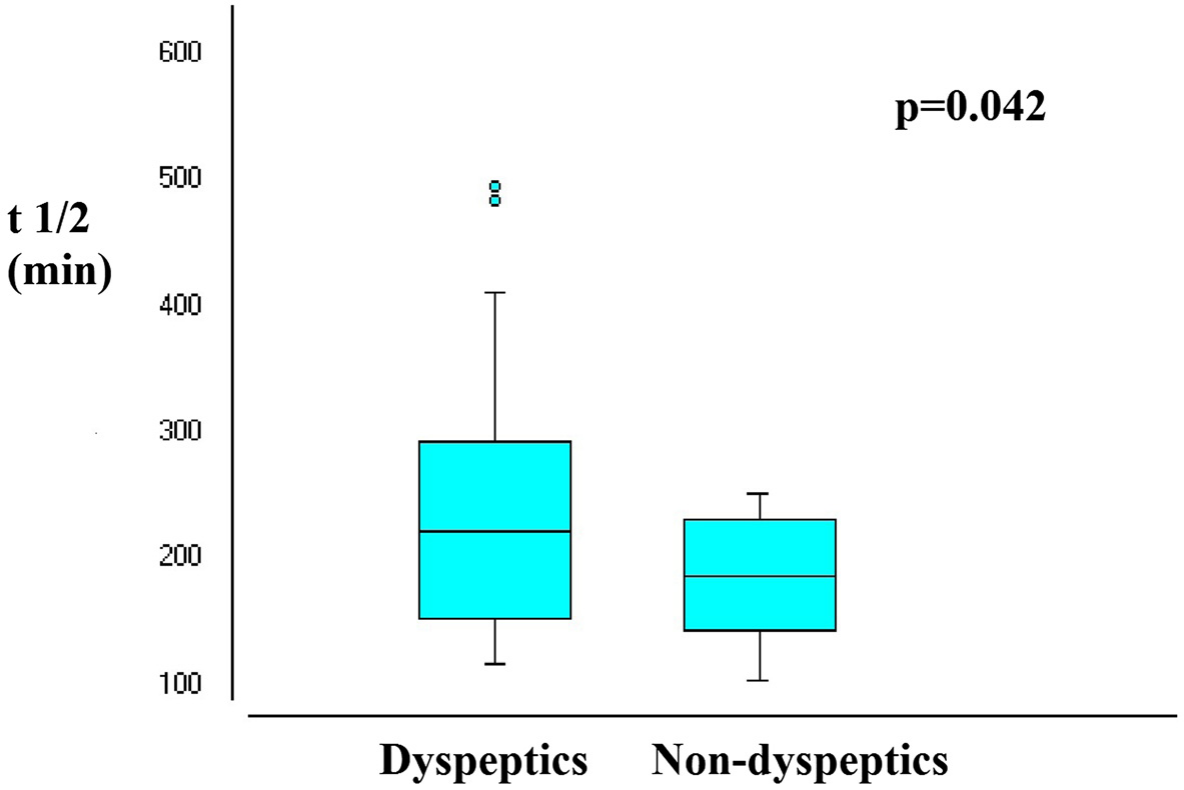
Comparison of t1/2 between dyspeptics and non-dyspeptics.

**Figure 2:**
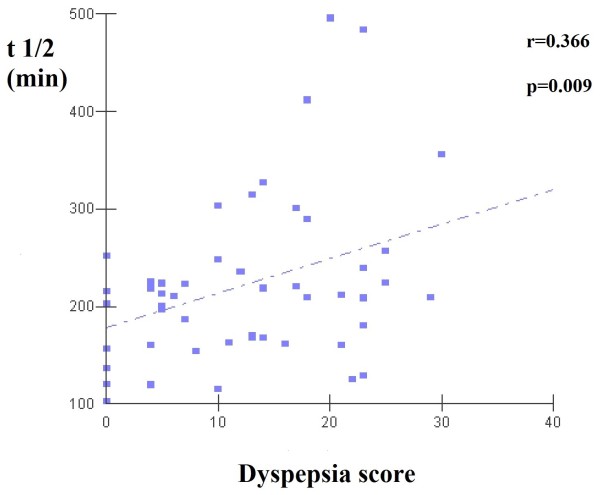
Correlation between t1/2 and dyspepsia scores.

Gastric emptying time estimated by t1/2 was different between dyspeptics and non-dyspeptics, respectively 238.0 ± 92.9 vs. 185.5 ± 45.5 minutes (p = 0.042) (Figure 1). According to the cut-off point of 200 minutes to classify gastric emptying delay, the mean t1/2 indicated gastric emptying delay among dyspeptics but not among non-dyspeptics. Additionally, the t1/2 was positively correlated with dyspepsia score (r = 0.366; p = 0.009) (Figure 2).

## Discussion

Our results confirmed the hypothesis that gastric emptying delay is associated with dyspepsia among ESRD patients on HD. First, we found longer gastric emptying time in dyspeptics compared to non-dyspeptics. Indeed, among dyspeptics, mean gastric emptying time was above the cut-off of 200 minutes, which classifies gastric emptying delay [14]. On the other hand, mean gastric emptying time was below 200 minutes in non-dyspeptics. Second, there was a positive correlation between gastric emptying time and dyspepsia score (higher score associated with more dyspeptic symptoms).

Due to its high prevalence and potential clinical importance, practitioners should pay attention to dyspepsia among the various ESRD patient symptoms. The prevalence of 68% in our study is within the range found in the literature, from 48% to 70% [3,4]. Dyspeptic symptoms can negatively affect quality of life and psychological well-being in the general population as well as in ESRD patients [5,17]. Awareness of the most common mechanisms involved in dyspepsia generation is crucial for successful management of dyspepsia. In theory, improvement of dyspepsia could ameliorate patients’ quality of life.

Unfortunately, gastric emptying delay, a suggested mechanism provoking dyspepsia, is difficult to assess in daily practice. The breath test we used is safe, but likely not suitable for larger samples due to the cost and need to spend extensive time for each evaluation.

Although we found a significant positive and linear correlation between t1/2 and dyspepsia score, we cannot ignore the overlap of gastric emptying time between the groups with and without dyspepsia (Figure 1). This is expected when studying dyspepsia, which is known to have multifactorial causes. Further studies are necessary to confirm gastric emptying delay as a main cause of dyspepsia among patients on HD and to formulate recommendations for appropriate treatment.

Even with a small size, our sample is larger compared to prior studies [18-20]. Indeed, to the best of our knowledge this study involves the first clinical sample of Brazilian HD patients submitted to an evaluation of gastric emptying rate using the ^13^carbon octanoic acid breath test. We are aware of limitations. First, the PAQYQ was validated in a group of Brazilian patients with nonulcer dyspepsia and healthy volunteers, but not specifically among ESRD patients. Second, it would be better to study diabetics and non-diabetics separately because diabetics are likely to present more gastroparesis than non-diabetics. However, our resources limited us to 50 experiments. Thus, random selection was used and generated a typical HD sample from underdeveloped regions with 25% diabetics. Third, only routine laboratory variables were evaluated. If we could test other laboratory variables such as gastrointestinal peptide and cytokines levels, it would help to elucidate the mechanisms involved in the relationship of dyspepsia, gastric emptying and renal failure.

As a preliminary study, we believe the clinical implications that must be highlighted are that practitioners should routinely search for symptoms of dyspepsia in HD patients. Dyspepsia is highly prevalent and easy to assess. Second, the treatment of dyspepsia could improve quality of life in HD patients. Third, the use of prokinetics in cases of proven functional dyspepsia, when other treatments of dyspeptic symptoms are not successful, will depend on further studies confirming the role of gastric emptying time as an independent cause of dyspepsia.

## Conclusions

Functional dyspepsia is very prevalent and is associated with gastric emptying delay. We assessed gastric emptying time by the octanoic acid breath test in a sample of ESRD on HD. Our findings raise the question about the role of prokinetics when the usual treatment of dyspeptic symptoms is not successful in proven functional dyspepsia cases. Future research is needed with larger samples, using a more workable method of measuring gastric emptying time.

## Abbreviations

CO2: Carbon dioxide; ESRD: End-stage renal disease; HD: Hemodialysis; PADYQ: Porto Alegre dyspeptic symptoms questionnaire; T1/2: Time in minutes to metabolize the first half of the ^13^carbon dose in the test meal.

## Competing interests

The authors declare that they have no competing interests.

## Authors’ contributions

LDSJ was responsible for the conception and design. PRS conducted analysis and interpretation of data. AAS and MHLPS edited and revised the manuscript. All authors read and approved the final manuscript.

## Pre-publication history

The pre-publication history for this paper can be accessed here:

http://www.biomedcentral.com/1471-2369/14/275/prepub
